# Cerebellar infarction and aneurysmal subarachnoid hemorrhage: An unusual presentation and rare complications of rhinocerebral mucormycosis

**Published:** 2015-10-07

**Authors:** Payam Sasannejad, Ali Ghabeli-Juibary, Samira Aminzadeh, Nahid Olfati

**Affiliations:** 1 Department of Neurology, School of Medicine, Mashhad University of Medical Sciences, Mashhad, Iran; 2 Department of Neurology, School of Medicine, Student Research Committee, Mashhad University of Medical Sciences, Mashhad, Iran

**Keywords:** Stroke, Mucormycosis, Subarachnoid Hemorrhage

The most common clinical presentation of mucormycosis is rhino-orbital-cerebral infection, which is supposed to begin with inhalation of spores into the paranasal sinuses of a vulnerable host. Hyperglycemia, with an associated metabolic acidosis, is the most common underlying state.^1^

The hallmarks of spread outside the sinuses are tissue necrosis of the palate.^1^^,^^2^

We present a 57-year-old man admitted to the emergency department of Ghaem Hospital affiliated to Mashhad University of Medical Sciences, Iran, with fever, decreased the level of consciousness, and left facial swelling from 1 week before admission. He was known the case of diabetes mellitus from 2 years before, and he was under treatment with oral hypoglycemic agents. Initiation of his symptoms was with a common cold and headache from 20 days before.

He was smoker and opiate addict. His drug history was glibenclamide, amlodipine, acyclovir ophthalmic drop and oral diazepam tablets. On physical examination, the patient was febrile (temperature: 39 °C). Ear nose and throat examination showed facial asymmetry due to complete ptosis, significant edema of the right eyelid, palatal necrosis and left alveolar ridge necrosis. On the left side of the nose middle and inferior conchae necrosis was observed. The right side of the nose was normal.

On mental status examination, he was drowsy but he was conscious and obeyed the commands. Examination of cranial nerves showed that he had multiple cranial nerve palsy. Fundoscopy was normal. The left eye had ptosis and pupil was mydriatic and non-reactive. Left frozen eye (3, 4, 6 cranial neuropathies) hypoesthesia of the left side of the face (V1, V2 of left fifth cranial neuropathy) left peripheral seventh nerve palsy (previous bell’s palsy). Other cranial nerves were normal. The motor examination was normal. Examination of sensory and gait of the patient was unremarkable because of his drowsiness. Ophthalmology and otolaryngology consult performed urgently.

The patient was started on intravenous amphotericin-B and wide spectrum antibiotics. Debridement of the left nasal cavity was subsequently performed. Biopsy samples obtained from the nasal eschar illustrated the picture of mucormycosis with some foci of non-septate fungal hyphae and hyphal branches typically at right angles.

The level of consciousness suddenly decreased 1 week after initiation of treatments. Computed tomography (CT) of the head showed extensive subarachnoid hemorrhage. The patient underwent emergent ventriculostomy and brain CT angiography performed 7 days after surgery. Brain CT angiography showed two consecutive fusiform aneurysms in a superior cerebellar artery (5.17 × 5.50 mm) and (4.17 × 5.55 mm). The patient finally died 2 months after admission in stroke intensive care unit (Figure 1).

Mucormycosis is a rare invasive fungal infection with a high rate of morbidity and mortality. Mucor organisms show aggressive characteristics regarding vascular and cranial nerve invasion and extension; thus, imaging and explanations of areas of involvement are key.^3^^,^^4^ In addition, rhinocerebral mucormycosis should be considered in the appropriate patient presenting with symptoms of unilateral cranial nerve involvement suggesting Garcin syndrome.^2^^,^^5^ In our patient, the ruptured aneurysm was diagnosed after a subarachnoid hemorrhage and the patient finally died after 2 months on March 26, 2014. The present case emphasizes an atypical presentation of fungal infection that can perplex any physician and thus delay diagnosis. Subarachnoid hemorrhage due to true mycotic aneurysmal rupture is consistently associated with a fatal outcome.^4^^,^^5^ Another aspect of this case was infarction of the cerebellum in the territory superior cerebellar artery at the first magnetic resonance imaging (MRI). This finding was misleading as an ischemic atherosclerotic infarction because of risk factors of the patient, but actually it was related to superior cerebellar artery aneurysm. This patient first treated for rhinocerebral mucormycosis but an aneurysm ruptured suddenly because of undiagnosed mycotic aneurysm, which was fatal for our patient. It is noteworthy to emphasize on an early angiographic study in patients with rhinocerebral mucormycosis and acute ischemic stroke on presentation because the delay in early diagnosis of a concealed aneurysm may be lethal for the patient.

In conclusion, recognition and early intervention of the underlying vascular complication that predispose patients to fatal outcome are critical in order to avoid serious complications of this rare infection especially when the acute infarction is evident in brain MRI.

**Figure 1:**
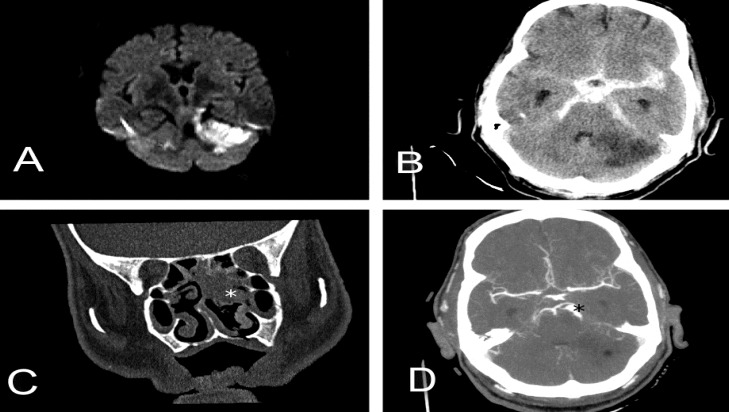
(A) Axial diffusion-weighted magnetic resonance imaging shows increased signal in left cerebellar hemisphere, representing acute ischemic infarction with restricted diffusion pattern, (B) axial computed tomography (CT) of the head shows extensive subarachnoid hemorrhage and cerebellar ischemic stroke, (C) CT coronal image shows an inflammatory process involving nasal cavity and ethmoid cells (white star), (D) Two consecutive fusiform aneurysms in superior cerebellar artery are evident in axial brain CT-angiography (black star).

## Notes:

**How to cite this article: **Sasannejad P, Ghabeli-Juibary A, Aminzadeh S, Olfati N. Cerebellar infarction and aneurysmal subarachnoid hemorrhage: An unusual presentation and rare complications of rhinocerebral mucormycosis. Iran J Neurol 2015; 14(4): 222-4.
